# Workplace factors associated with mental health of healthcare workers during the COVID-19 pandemic: an international cross-sectional study

**DOI:** 10.1186/s12913-021-06279-6

**Published:** 2021-03-21

**Authors:** Ankur Khajuria, Wojtek Tomaszewski, Zhongchun Liu, Jian-hua Chen, Roshana Mehdian, Simon Fleming, Stella Vig, Mike J. Crawford

**Affiliations:** 1 Kellogg College, University of Oxford, Oxford, UK; 2 Royal College of Surgeons of England, London, UK; 3 Department of Surgery and Cancer, Imperial College London, London, UK; 4 Institute for Social Science Research, University of Queensland, Brisbane, Australia; 5 Department of Psychiatry, Renmin Hospital, Wuhan University, Wuhan, China; 6 Shanghai Clinical Research Centre for Mental Health, Shanghai Jiao Tong University School of Medicine, Shanghai, China; 7 Institute of Health Sciences Education, Queen Mary University of London, London, UK; 8 Division of Psychiatry, Imperial College London, London, UK

**Keywords:** Mental health, COVID-19, Coronavirus, Workplace, Healthcare workers

## Abstract

**Background:**

The association of workplace factors on mental health of healthcare workers (HCWs) during the COVID-19 pandemic needs to be urgently established. This will enable governments and policy-makers to make evidence-based decisions. This international study reports the association between workplace factors and the mental health of HCWs during the pandemic.

**Methods:**

An international, cross-sectional study was conducted in 41 countries. The primary outcome was depressive symptoms, derived from the validated Patient Health Questionnaire-2 (PHQ-2). Multivariable logistic regression identified factors associated with mental health outcomes. Inter-country differences were also evaluated.

**Results:**

A total of 2527 responses were received, from 41 countries, including China (*n* = 1213; 48.0%), UK (*n* = 891; 35.3%), and USA (*n* = 252; 10.0%). Of all participants, 1343 (57.1%) were aged 26 to 40 years, and 2021 (80.0%) were female; 874 (34.6%) were doctors, and 1367 (54.1%) were nurses. Factors associated with an increased likelihood of depressive symptoms were: working in the UK (OR = 3.63; CI = [2.90–4.54]; *p* < 0.001) and USA (OR = 4.10; CI = [3.03–5.54]), *p* < 0.001); being female (OR = 1.74; CI = [1.42–2.13]; *p* < 0.001); being a nurse (OR = 1.64; CI = [1.34–2.01]; *p* < 0.001); and caring for a COVID-19 positive patient who subsequently died (OR = 1.20; CI = [1.01–1.43]; *p* = 0.040). Workplace factors associated with depressive symptoms were: redeployment to Intensive Care Unit (ICU) (OR = 1.67; CI = [1.14–2.46]; *p* = 0.009); redeployment with perceived unsatisfactory training (OR = 1.67; CI = [1.32–2.11]; *p* < 0.001); not being issued with appropriate personal protective equipment (PPE) (OR = 2.49; CI = [2.03–3.04]; *p* < 0.001); perceived poor workplace support within area/specialty (OR = 2.49; CI = [2.03–3.04]; *p* < 0.001); and perceived poor mental health support (OR = 1.63; CI = [1.38–1.92]; *p* < 0.001).

**Conclusion:**

This is the first international study, demonstrating that workplace factors, including PPE availability, staff training pre-redeployment, and provision of mental health support, are significantly associated with mental health during COVID-19. Governments, policy-makers and other stakeholders need to ensure provision of these to safeguard HCWs’ mental health, for future waves and other pandemics.

**Supplementary Information:**

The online version contains supplementary material available at 10.1186/s12913-021-06279-6.

## Introduction

Severe acute respiratory syndrome coronavirus (SARS-CoV-2) has now infected over 100 million people, with more than 2,000,000 deaths globally [1]. The World Health Organisation (WHO) declared the novel coronavirus disease 2019 (COVID-19), a pandemic on March 11, 2020 [2]. Health systems have rightly prioritized testing, critical patient care and reducing viral transmission and the reproduction rate (R). However, concerns have also been raised about the impact of COVID-19 on mental health, especially among Healthcare workers (HCWs) [3–6]. This is especially pertinent given the pre-existing mental health crisis amongst HCWs, with high rates of stress-related psychiatric illness and reliance on smoking, alcohol, drugs and self-medication as coping mechanisms [7]. HCWs also represent a particularly vulnerable group during pandemics, due to the high risk of infection, fear of contagion and spread to family members and increased work-related stressors, including the need to make life-prioritizing decisions [8].

The detrimental impact of previous pandemics on mental health of HCWs has been well documented [9–11]. Studies from the 2003 SARS pandemic reported higher levels of depression [12], anxiety [13], post-traumatic stress disorder (PTSD) [14], burnout [15] and stress [10] amongst HCWs, with symptoms persisting up to 1 year after the pandemic [10] and nursing staff reporting the worst outcomes [16]. Consequently, similar concerns for HCWs treating COVID-19 have arisen. Unsurprisingly, in a Chinese study of 1257 HCWs treating COVID-19 patients in Wuhan, China, a considerable proportion reported depressive symptoms, anxiety and distress, with nurses, women and frontline workers reporting more severe symptoms [17]. A systematic review of 13 studies, involving responses from 33,062 Chinese and Singaporean HCWs during the COVID-19 pandemic, reported a pooled prevalence of depression at 22.8% [4].

Nevertheless, the studies did not explore the relative contribution of key workplace factors, including perceived adequacy of personal protective equipment (PPE) [18], adequacy/satisfaction with training prior to redeployment [15], level of mental health support in the workplace [19] and perceived support in the area of work [20]. Perceived adequacy of training and support, provision of workplace mental health support and perceived adequacy of PPE were shown to be protective factors for depression and PTSD for HCWs in the SARS pandemic [15, 18, 19]. Current published literature during the COVID-19 pandemic is limited by poor external validity and generalizability to countries outside Asia. Moreover, no study has evaluated inter-country differences, which may influence mental health. These include: 1) different countries being at different stages of the pandemic (e.g. pre- and post-peak); 2) different country-level responses and messaging around the danger of COVID-19 [21]; and 3) underlying cultural differences in absorbing negative emotions and not experiencing distress [22].

The impact of COVID-19 on mental health and wellbeing of HCWs outside of Asia needs to be urgently established to enable governments and policy-makers to make evidence-based decisions, and subsequently employ targeted strategies. We hypothesize that the pandemic is associated with a detrimental impact on mental health of HCWs and aim to identify key correlates of mental health, in 2527 HCWs from 41 countries, including China, the United Kingdom (UK) and USA.

## Methods

### Study design

This study followed the American Association for Public Opinion Research (AAPOR) reporting guidance [23]. Between 18 April 2020 and 24 May 2020, an international cross-sectional study was conducted in 41 countries, including China, UK and USA. The survey was translated in Mandarin by Chinese authors (ZL and JC) prior to dissemination in China. Non-probability, convenience sampling was employed, using online social media and professional networking platforms, *Twitter*, *LinkedIn*, *Facebook and WeChat*. Participation was entirely voluntary and participants were allowed to terminate the survey at any time they desired. Confidentiality and privacy were protected, adhering to the General Data Protection Regulation (GDPR). All survey responses were entirely anonymous. According to advice obtained from the NHS Health Research Authority’s online decision tool [24], the study did not require formal ethics approval.

### Sample size

Power analysis was conducted to estimate the sample size needed for multi-group comparisons of mental health. The baseline level of depressive symptoms among HCWs was set at 35% in line with a previous study of the SARS outbreak [10]. Power analysis, using statistical package Stata (Stata Corp) version 16.0, was performed assuming significance level α = 0.05 and the power of 0.8 (β = 0.2). We calculated that a sample of at least 1128 was needed to detect a 10% difference in levels of depressive symptoms across three groups (e.g. three countries or age groups), with at least 376 responses per group (e.g. country/age group), or at least 170 per group (e.g. country/age group) to detect a 15% difference.

### Outcomes, correlates and covariates

The questionnaire consisted of two main sections. Section 1 comprised demographic data, including age (coded as 18–25; 26–33; 34–40; 41–48; 49+), gender (male, female), occupation/role (coded as Doctors; Nurses; and Other – comprising allied healthcare professionals, interns, and hospital management/administrative staff) and country of origin (coded as China, UK, US, Other) and whether they live alone or with others. Responses were also obtained on whether participants had personally been involved in care of COVID-19 positive patients who died, if they had been redeployed to a different specialty and how satisfied they felt with the amount and quality of training prior to their new roles.

Section 2 comprised assessment of mental health. The primary outcome was symptoms of depression, assessed using a single question ‘*During this outbreak, have you felt down, depressed, or hopeless?*’ This question was derived from one of two items in the validated Patient Health Questionnaire-2 (PHQ-2) depression scale [25], and other widely used questionnaires for screening for depression across medical and occupational health settings [26]. Data was also collected on key workplace correlates of mental health. These included: perceived adequacy of PPE; provision of mental health support; and, perceived level of support within the clinical role. The questions utilized in the survey are listed in Supplement 1.

### Statistical analysis

Data analysis was performed using statistical software Stata (Stata Corp), version 16.0. The significance level was set at α = 0.05, with all tests 2-tailed. Given the ordinal nature of the outcome variable, Kruskal-Wallis H Test was used to determine differences in the frequency of depressive symptoms across groups determined by categories of covariates (most of which are multi-categorical). Ordered logistic regression (ologit in Stata) was used to determine risk factors for depressive symptoms, while adjusting for potentially confounding relationships between covariates. Factors included in the models were selected based on hypothesized relationships with depressive symptoms and previous studies, and included: country, gender, age, living arrangements, role, having been involved in care of person who died of COVID-19, redeployment: role and training received prior to redeployment; perceived risk associated with PPE, workplace support in the area/specialty, and mental health support at workplace. The results from these multivariable analyses are presented as proportional odds ratios (ORs) with 95% CIs. Analyses were first run on the overall sample (while adjusting for country effects), with a final step involving fitting models using individual country samples separately for the three largest countries in our data (UK, US and China). Wald Chi-Squared Test was used to formally test for the differences in coefficients across the countries.

### Patient and public involvement

Patients and the public were not involved in design, conduct, reporting, or dissemination plans of our research. Our study author team, comprising healthcare workers, was involved in the design, conduct and reporting of the research.

### Reporting

The study has been reported as per the STROBE checklist (Supplement 2).

## Results

### Sample characteristics

A total of 2527 responses were received, covering respondents from 41 countries. All respondents provided answers to all questions, resulting in no missing data. The largest number of responses came from China (*n* = 1213; 48.0%), followed by the UK (*n* = 891; 35.3%), and the USA (*n* = 252; 10.0%), with the other countries comprising 171 (6.8%) responses (Table 1). These included, 53 (2.1%) responses from Europe; 55 (2.1%) responses from Oceania; 31 (1.2%) from Asia; 15 (0.60%) from North America; 11 (0.4%) from South America; and 6 (0.2%) from Africa.

**Table 1:** Demographic and occupational characteristics by country

	China		UK		US		Other		Total	
	N	%	N	%	N	%	N	%	N	%
**Overall**	1213	100.0	891	100.0	252	100.0	171	100.0	2527	100.0
**Gender**										
*Male*	171	14.1	242	27.2	43	17.1	50	29.2	506	20.0
*Female*	1042	85.9	649	72.8	209	82.9	121	70.8	2021	80.0
**Age**										
*18–25*	188	15.5	72	8.1	7	2.8	8	4.7	275	10.9
*26–33*	525	43.3	307	34.5	43	17.1	41	24	916	36.2
*34–40*	226	18.6	202	22.7	62	24.6	37	21.6	527	20.9
*41–48*	179	14.8	175	19.6	67	26.6	43	25.1	464	18.4
*49+*	95	7.8	135	15.2	73	29.0	42	24.6	345	13.7
**Living arrangements**										
*Lives with others*	1120	92.3	756	84.8	201	79.8	147	86.0	2224	88.0
*Lives alone*	93	7.7	135	15.2	51	20.2	24	14.0	303	12.0
**Role**										
*Doctors*	165	13.6	496	55.7	114	45.2	99	57.9	874	34.6
*Nurses*	993	81.9	244	27.4	97	38.5	33	19.3	1367	54.1
*Other*	55	4.5	151	16.9	41	16.3	39	22.8	286	11.3
**Involved in care of person who died of COVID-19**										
*No*	1002	82.6	401	45	117	46.4	137	80.1	1657	65.6
*Yes*	211	17.4	490	55	135	53.6	34	19.9	870	34.4
**Redeployment**										
*Not redeployed*	623	51.4	557	62.5	169	67.1	129	75.4	1478	58.5
*General medicine*	51	4.2	71	8.0	15	6.0	5	2.9	142	5.6
*Emergency medicine*	49	4	29	3.3	6	2.4	7	4.1	91	3.6
*Intensive Care Unit*	59	4.9	168	18.9	45	17.9	15	8.8	287	11.4
*Other*	431	35.5	66	7.4	17	6.7	15	8.8	529	20.9
**Redeployment: training received** *(of those redeployed)*										
*No training/ not satisfied*	248	42.0	199	59.6	54	65.1	22	52.4	523	49.9
*Satisfied with training*	342	58.0	135	40.4	29	34.9	20	47.6	526	50.1
**PPE: perceived risk**										
*Low*	1025	84.5	707	79.3	191	75.8	136	79.5	2059	81.5
*High*	188	15.5	184	20.7	61	24.2	35	20.5	468	18.5
**Support in area/ specialty**										
*Adequate*	1084	89.4	732	82.2	166	65.9	120	70.2	2102	83.2
*Poor*	129	10.6	159	17.8	86	34.1	51	29.8	425	16.8
**Mental health support**										
*Adequate*	853	70.3	476	53.4	115	45.6	63	36.8	1507	59.6
*Poor*	360	29.7	415	46.6	137	54.4	108	63.2	1020	40.4

‘Other’ roles include allied healthcare professionals, interns, and hospital management/administrative staff; ‘Other’ redeployment includes contingency planning and telehealth

*ICU* Intensive Care Unit, *PPE* Personal Protective Equipment

In the overall sample, most participants were female (*n* = 2021; 80.0%) and aged 26 to 40 years (*n* = 1343; 57.1%). A total of 874 participants across all countries (34.6%) were doctors, while 1367 (54.1%) were nurses and 286 (11.3%) were in other roles, such as allied healthcare professionals, interns, and hospital management/administrative staff. In total, 870 (34.4%) participants had been personally involved in care for somebody who died of COVID-19; and a total of 1049 (41.5%) had been redeployed during the COVID-19 pandemic, including 287 (11.4%) redeployed to ICU, 142 (5.6%) redeployed to General Medicine, 91 (3.6%) to Emergency Medicine and 529 (20.9%) were redeployed to other roles, including contingency planning and telehealth. Among those redeployed, 526 (50.1% of the redeployed) were satisfied with training they received prior to redeployment. Most participants in the sample perceived the risk associated with PPE they had been issued as low (*n* = 2059; 81.5%), felt adequately supported in their areas or specialty (*n* = 2102; 83.2%), and said they received adequate mental health support in the workplace (*n* = 1507; 59.6%).

### Bivariate associations

Table 2 shows the distribution of the outcome variable by categories of key demographic and occupational characteristics (as per Table 1), as well as factors describing the level of training and support received at workplace. Based on Kruskal-Wallis H test, there are cross-national differences in the sample (*p* < 0.001) in the frequency of reporting depressive symptoms. The proportion of those reporting depressive symptoms ‘all of the time’ or ‘often’ was much lower in China (*n* = 73; 6%) than in the UK (*n* = 266; 29.9%), the USA (*n* = 81; 32.2%) or other countries (*n* = 33; 19.3%). There are also significant associations between the frequency of depressive symptoms and demographic factors, including gender (*p* < 0.001), age (*p* = 0.013), and living alone (*p* = 0.004). Bivariate analyses further indicate differences in the frequency of reporting depressive symptoms and role (*p* < 0.001), having been involved in care for somebody who died of COVID-19 (*p* < 0.001), and factors associated with redeployment, including area of redeployment (*p* < 0.001), and satisfaction with training provided prior to redeployment (*p* < 0.001).

**Table 2:** Frequency of symptoms of depression by categories of covariates

	*None of the time*		*Rarely*		*Some of the time*		*Often*		*All the time*		*Total*		K-W test (chi sq (df), ***p***-value)
	N	*%*	N	*%*	N	*%*	N	*%*	N	*%*	N	*%*	
**Total**	349	*13.8*	603	*23.9*	1122	*44.4*	385	*15.2*	68	*2.7*	2527	*100*	
**Country**													
*China*	247	*20.4*	347	*28.6*	546	*45.0*	68	*5.6*	5	*0.4*	1213	*100*	259.749 (3) < 0.001
*UK*	79	*8.9*	180	*20.2*	366	*41.1*	221	*24.8*	45	*5.1*	891	*100*	
*US*	8	*3.2*	36	*14.3*	127	*50.4*	70	*27.8*	11	*4.4*	252	*100*	
*Other*	15	*8.8*	40	*23.4*	83	*48.5*	26	*15.2*	7	*4.1*	171	*100*	
**Sex**													
*Male*	106	*21.0*	123	*24.3*	197	*38.9*	70	*13.8*	10	*2.0*	506	*100*	18.209 (1) < 0.001
*Female*	243	*12.0*	480	*23.8*	925	*45.8*	315	*15.6*	58	*2.9*	2021	*100*	
**Age**													
*18–25*	44	*16.0*	80	*29.1*	112	*40.7*	29	*10.6*	10	*3.6*	275	*100*	12.688 (4) *p* = 0.013
*26–33*	124	*13.5*	219	*23.9*	418	*45.6*	138	*15.1*	17	*1.9*	916	*100*	
*34–40*	57	*10.8*	116	*22.0*	246	*46.7*	91	*17.3*	17	*3.2*	527	*100*	
*41–48*	69	*14.9*	116	*25.0*	191	*41.2*	76	*16.4*	12	*2.6*	464	*100*	
*49+*	55	*15.9*	72	*20.9*	155	*44.9*	51	*14.8*	12	*3.5*	345	*100*	
**Living arrangements**													
*Lives with others*	313	*14.1*	547	*24.6*	980	*44.1*	322	*14.5*	62	*2.8*	2224	*100*	8.316 (1) *p* = 0.004
*Lives alone*	36	*11.9*	56	*18.5*	142	*46.9*	63	*20.8*	6	*2.0*	303	*100*	
**Role**													
*Doctors*	122	*14.0*	208	*23.8*	373	*42.7*	144	*16.5*	27	*3.1*	874	*100*	20.020 (2) < 0.001
*Nurses*	201	*14.7*	340	*24.9*	616	*45.1*	177	*13.0*	33	*2.4*	1367	*100*	
*Other*	26	*9.1*	55	*19.2*	133	*46.5*	64	*22.4*	8	*2.8*	286	*100*	
**Involved in care of person who died of COVID-19**													
*No*	268	*16.2*	424	*25.6*	743	*44.8*	194	*11.7*	28	*1.7*	1657	*100*	69.613 (1) < 0.001
*Yes*	81	*9.3*	179	*20.6*	379	*43.6*	191	*22.0*	40	*4.6*	870	*100*	
**Redeployment**													
*Not redeployed*	215	*14.6*	353	*23.9*	644	*43.6*	230	*15.6*	36	*2.4*	1478	*100*	58.221 (4) < 0.001
**Redeployment: area**													
*General medicine*	26	*6.9*	75	*19.8*	169	*44.7*	90	*23.8*	18	*4.8*	378	*100*	
*Emergency medicine*	11	*12.1*	23	*25.3*	35	*38.5*	20	*22.0*	2	*2.2*	91	*100*	
*ICU*	15	*5.2*	52	*18.1*	134	*46.7*	70	*24.4*	16	*5.6*	287	*100*	
*Other*	92	*17.4*	135	*25.5*	246	*46.5*	46	*8.7*	10	*1.9*	529	*100*	
**Redeployment: training received**													77.287 (2) < 0.001
*No training/ not satisfied*	46	*8.8*	90	*17.2*	245	*46.9*	112	*21.4*	30	*5.7*	523	*100*	
*Satisfied with training*	88	*16.7*	160	*30.4*	233	*44.3*	43	*8.2*	2	*0.4*	526	*100*	
**PPE: perceived risk**													
Low	323	*15.7*	534	*25.9*	919	*44.6*	255	*12.4*	28	*1.4*	2059	*100*	140.574 (1) < 0.001
High	26	*5.6*	69	*14.7*	203	*43.4*	130	*27.8*	40	*8.6*	468	*100*	
**Support in area/ specialty**													
Adequate	328	*15.6*	557	*26.5*	929	*44.2*	260	*12.4*	28	*1.3*	2102	*100*	175.358 (1) < 0.001
Poor	21	*4.9*	46	*10.8*	193	*45.4*	125	*29.4*	40	*9.4*	425	*100*	
**Mental health support**													
Adequate	253	*16.8*	420	*27.9*	669	*44.4*	142	*9.4*	23	*1.5*	1507	*100*	132.022 (1) < 0.001
Poor	96	*9.4*	183	*17.9*	453	*44.4*	243	*23.8*	45	*4.4*	1020	*100*	

‘Other’ roles include allied healthcare professionals, interns, and hospital management/administrative staff; ‘Other’ redeployment includes contingency planning and telehealth

*K-W test* Kruskal-Wallis H Test, *chi sq* Chis squared statistic, *df* Degrees of freedom, *ICU* Intensive Care Unit, *PPE* Personal Protective Equipment

Finally, there were significant bivariate associations between the frequency of depressive symptoms and feeling psychologically at risk by not being issued appropriate PPE (*p* < 0.001), reported poor support in the workplace in the respondent’s area/specialty (*p* < 0.001) and reported poor metal health support during the COVID-19 outbreak (*p* < 0.001).

### Multivariable regression models

Ordered logistic regression model was fitted to determine risk factors for depressive symptoms (with frequency measured using the five response categories on the outcome variable), while adjusting for potentially confounding relationships between covariates (Table 3).

**Table 3:** Results from ordered logistic regression model (proportional odds ratios), overall model

	Odds Ratio	Std. Err.		[95% Conf. Interval]		***P*** value	Overall ***p***
**Country**							
*China*	1.00					(ref)	
*UK*	3.63	0.41		2.90	4.54	< 0.001	
*US*	4.10	0.63		3.03	5.54	< 0.001	< 0.001
*Other*	2.49	0.43		1.77	3.50	< 0.001	
**Gender**							
*Male*	1.00					(ref)	
*Female*	1.74	0.18		1.42	2.13	< 0.001	< 0.001
**Age**							
*18–25*	1.00					(ref)	
*26–33*	1.07	0.14		0.83	1.38	0.598	
*34–40*	1.24	0.18		0.94	1.65	0.132	0.0025
*41–48*	0.84	0.12		0.63	1.12	0.241	
*49+*	0.79	0.13		0.58	1.08	0.145	
**Living arrangements**							
*Living with others*	1.00					(ref)	
*Living alone*	1.07	0.13		0.85	1.35	0.547	0.547
**Role**							
*Doctors*	1.00					(ref)	
*Nurses*	1.64	0.17		1.34	2.01	< 0.001	< 0.001
*Other*	1.68	0.22		1.30	2.18	< 0.001	
**Involved in care of person who died of COVID-19**							
*No*	1.00					(ref)	
*Yes*	1.20	0.11		1.01	1.43	0.040	0.040
**Redeployment - role**							
*General medicine*	1.00					(ref)	
*Emergency medicine*	1.52	0.39		0.92	2.50	0.100	
*ICU*	1.67	0.33		1.14	2.46	0.009	
*Other*	1.28	0.23		0.90	1.83	0.169	0.065
*Not redeployed*	1.48	0.26		1.06	2.09	0.023	
**Redeployment - training received**							
*Satisfactory training*	1.00					(ref)	
*No training/ not satisfactory training*	1.67	0.20		1.32	2.11	< 0.001	< 0.001
**PPE: perceived risk**							
Low	1.00					(ref)	
High	2.49	0.26		2.03	3.05	< 0.001	< 0.001
**Support in area/ specialty**							
Adequate	1.00					(ref)	
Poor	2.24	0.25		1.79	2.80	< 0.001	< 0.001
**Mental health support**							
Adequate	1.00					(ref)	
Poor	1.63	0.14		1.38	1.92	< 0.001	< 0.001
/cut1	0.31	0.23		−0.14	0.75		
/cut2	1.81	0.23		1.36	2.25		
/cut3	4.28	0.24		3.81	4.75		
/cut4	6.62	0.27		6.09	7.16		
N			2527				

‘Other’ roles include allied healthcare professionals, interns, and hospital management/administrative staff; ‘Other’ redeployment includes contingency planning and telehealth

*Std. Err.* Standard error, *Conf. Interval* Confidence interval, *ICU* Intensive Care Unit, *PPE* Personal Protective Equipment

Factors significantly associated with increased likelihood (expressed as proportional ORs) of more frequent depressive symptoms, include: working in the UK (OR = 3.63; CI = [2.90–4.54]; *p* < 0.001) and the USA (OR = 4.10; CI = [3.03–5.54]), *p* < 0.001), being a female (OR = 1.74; CI = [1.42–2.13]; *p* < 0.001); being a nurse (OR = 1.64; CI = [1.34–2.01]; *p* < 0.001); having been involved in care of a COVID-19 positive patient who died (OR = 1.20; CI = [1.01–1.43]; *p* = 0.040), and having been redeployed to ICU (OR = 1.67; CI = [1.14–2.46]; *p* = 0.009).

Factors related to poor training and poor support in the workplace were all highly significant correlates of more frequent depressive symptoms, including having been redeployed without training or with unsatisfactory training (OR = 1.67; CI = [1.32–2.11]; *p* < 0.001), feeling at risk psychologically due to not being issued appropriate PPE (OR = 2.49; CI = [2.03–3.04]; *p* < 0.001), perceived poor support at workplace in the respondent’s area/specialty (OR = 2.49; CI = [2.03–3.04]; *p* < 0.001), and perceived poor mental health support (OR = 1.63; CI = [1.38–1.92]; *p* < 0.001).

### Cross-national patterns

The model was re-estimated on individual country samples, restricted to the three countries with largest samples: UK, US, and China (Table 4). Pos-hoc power analysis has confirmed that despite relatively low sample size for US, cross-national comparisons are adequately powered (power > 0.8) due to large sample sizes for UK and China. Results show patterns broadly consistent with the overall results. Specifically, being a female was associated with increased frequency of depressive symptoms in China (OR = 2.04; CI = [1.45–2.88]; *p* < 0.001) and in the UK (OR = 1.71; CI = [1.28–2.30]; *p* < 0.001). Being a nurse was significantly associated with more frequent reporting of depressive symptoms in the UK (OR = 2.61; CI = [1.88–3.62]; *p* < 0.001) but not in other countries; this association is statistically different across countries based on the Wald Chi-Squared Test (chi2[6]=25.24; *p* = < 0.001), suggesting that nurses face a higher risk of having depressive symptoms in the UK, compared with other countries. Perceived inadequacy of PPE was significantly associated with increased risk of having depressive symptoms more frequently in China (OR = 2.24; CI = [1.63,3.07]; *p* < 0.001), UK (OR = 3.39; CI = [2.42,4.75]; *p* < 0.001) and in the USA (OR = 2.00; CI = [1.09,3.68]; *p* = 0.024); the cross-national differences in the strength of this effect are statistically significant (chi2[3]=13.34; *p* = 0.004). Perceived poor support at workplace in the respondent’s area/specialty was associated with more frequent reporting of depressive symptoms in China (OR = 2.38; CI = [1.60,3.54]; *p* < 0.001) and in the UK (OR = 2.41; CI = [1.66,3.50]; *p* < 0.001). Perceived poor mental health support was also significantly associated with an increased frequency of depressive symptoms in China (OR = 1.56; CI = [1.21,2.02], *p* < 0.001) and in the UK (OR = 1.57; CI = [1.21,2.05]; *p* < 0.001).

**Table 4:** Results from ordered logistic regression model (proportional odds ratios), within-country models

	China	UK	US
**Gender**			
Male (ref)	1.00	1.00	1.00
Female	2.04^***^ [1.45,2.88]	1.71^***^ [1.28,2.30]	1.08 [0.53,2.19]
**Role**			
*Doctors (ref)*	1.00	1.00	1.00
*Nurses*	1.03 [0.73,1.46]	2.61^***^ [1.88,3.62]	1.49 [0.79,2.81]
*Other*	1.39 [0.76,2.54]	1.69^**^ [1.20,2.40]	3.34^**^ [1.55,7.23]
**PPE: perceived risk**			
Low (ref)	1.00	1.00	1.00
High	2.24^***^ [1.63,3.07]	3.39^***^ [2.42,4.75]	2.00^*^ [1.09,3.68]
**Support in area/ specialty**			
Adequate (ref)	1.00	1.00	1.00
Poor	2.38^***^ [1.60,3.54]	2.41^***^ [1.66,3.50]	1.68 [0.96,2.95]
**Mental health support**			
Adequate (ref)	1.00	1.00	1.00
Poor	1.56^***^ [1.21,2.02]	1.57^***^ [1.21,2.05]	1.59 [0.95,2.66]
N	1213	891	252

95% confidence intervals in brackets; ^*^ *p* < 0.05, ^**^ *p* < 0.01, ^***^ *p* < 0.001; models adjust for age, living arrangements, experience of involvement in care for somebody who died of COVID-19, and redeployment (role and satisfaction with training); *PPE* Personal Protective Equipment; ‘Other’ roles include allied healthcare professionals, interns, and hospital management/administrative staff

## Discussion

Data from over 2500 healthcare staff working in 41 countries from across the world reveals high levels of emotional distress with more than 60% reporting feeling down, depressed or hopeless at least some of the time, and one in six experiencing these feelings often or all the time. Levels of emotional distress were even higher among respondents from the UK and USA, where almost a third of respondents reported regular depressive thoughts. Higher levels of emotional distress were reported by women, by nurses and by those treating patients with COVID-19. Workplace factors also influenced the likelihood that staff reported poor mental health. People who stated that they had not been issued appropriate PPE were more than twice as likely to report depressive thoughts. Staff who were redeployed to Intensive Care Units and other emergency medical services were more likely to report poor mental health, especially those that did not feel satisfied with the amount or quality of training they received for their new role. Staff who felt they were well supported in the area they were working were less likely to report depressive thoughts, as were those who stated that they had received mental wellbeing support during the crisis.

Our findings are based on the largest international cross-sectional study of mental health of HCWs conducted since the start of the COVID-19 pandemic. Large numbers of responders from China, the UK and the USA mean that we have been able to explore differences in risk factors for poor mental health between these countries. By focusing on factors that services/institutions can change, such as availability of PPE and mental health support, we set out to generate data which can guide service delivery in countries which are at an earlier stage of exposure to the virus and help ensure that preparations are made now to protect the metal health of HCWs in future outbreaks of COVID-19 or other infectious diseases.

The results of this large international cross-sectional study provide support for findings from previous studies conducted solely in China and South East Asia [4, 17]. In keeping with surveys conducted among the general population, we found that women were more likely to report depressive symptoms than men [27]. We also found evidence of poorer mental health among nurses compared to doctors and among front-line workers who had treated patients with COVID-19 compared to those who had not, in line with previous studies [4, 17]. The main focus of this cross-sectional study was on workplace factors that are amenable to change. Our findings that, across different countries, access to PPE, training given to support those who are redeployed and wellbeing and mental health support influence the likelihood that front-line staff experience poor mental health are important.

In a survey of 304 HCWs in Iran, conducted in April 2020, Zhang and colleagues reported that staff who had better access to PPE were less likely to be mentally distressed [18]. Since then repeated concerns have been raised about access to PPE across the world [28, 29]. These results are in keeping with previous research which has highlighted how the psychosocial safety climate in which people work can have a direct bearing on their mental health [30].

Our finding, that access to PPE was associated with self-reported mental health among HCWs internationally, highlights the importance of PPE for protecting the mental health of HCWs. Steps, such as stockpiling PPE and having systems in place to produce PPE locally if needed, need to be taken to protect the mental as well as the physical health of front-line staff in the future [31].

Redeployment of HCWs has been an integral part of the response that health services have undertaken, to ensure that key areas are adequately staffed and patients with COVID-19 receive the care they need [32]. Our data shows that HCWs redeployed to critical care settings are at increased risk of depressive symptoms. Almost half of those who took part in this survey had been redeployed, and half of them stated that they were dissatisfied with the amount and quality of training they received for their new role. Our data shows that the quality of training that staff receive prior to redeployment influences their mental health and steps to improve training for such staff is also an important part of safeguarding the mental health of front-line HCWs.

HCWs who took part in this survey who stated they had received mental wellbeing support during the COVID-19 outbreak were less likely to have experienced feeling down, depressed or hopeless. Efforts to protect the mental health of front-line staff during the pandemic must ensure that they have access to this support.

The study has a number of important limitations. As a cross-sectional study, we do not know if the associations identified are causal. For instance, it is possible that HCWs who experience depressive symptoms may be more likely to state that support for their mental health was poor. As an online cross-sectional study of willing participants, it is unclear how representative those who took part in the survey are of all HCWs in these countries. It is possible that those who chose to take part had been more affected by the pandemic than those who did not. Our data does not therefore provide a reliable estimate of the proportion of HCWs in each country who were experiencing depressive thoughts, at the time the study was conducted. However, the aim of our study was to examine factors that may increase the likelihood of mental distress among frontline HCWs, and the data we collected has helped to identify these factors.

In an attempt to maximise the response rate we did not make a detailed assessment of respondents’ mental health. Instead, we used a single item question that was derived from the Patient Health Questionnaire-2 (PHQ-2), a widely used measure of mental health [25, 26]. While single item questions such as the one we used provide an indication of the likelihood that someone has poor mental health [33], they are less reliable than longer instruments [34]. The data we present should therefore not be viewed as providing a reliable measure of the proportion of people in the survey who had depression or other common mental disorders. Large number of responders from China, UK and the USA meant that we have been able to explore differences in risk factors for poor mental health between these countries. However the number of responses from other countries was insufficient to examine differences in risk factors for poor mental health beyond these countries. While the survey was conducted at the same time across all countries (mid-April to mid-May 2020), countries were going through different phases of the pandemic during this period, and this may go some way to explaining the differences we found between countries, with higher levels of emotional distress reported by staff working in the UK and USA compared to those working in China.

Cross-sectional surveys provide a rapid and efficient way to identify health needs and generate suggestions for interventions that may reduce them. Longitudinal studies are now needed to examine the course and impact of mental distress among HCWs. Prospective interventional studies will also be important in testing the impact of interventions that aim to reduce mental distress of frontline workers [5].

In conclusion, we have found that across the world, workplace factors, including of availability of PPE, training for staff who are redeployed, and provision of well-being and mental health support have a significant association with the likelihood that staff experience depressive symptoms. Efforts to increase pandemic preparedness need to ensure that provision of these and other resources that safeguard the mental health of front-line workers are in place, in order to ensure that services are better prepared for future waves of COVID-19 or other pandemics.

## Supplementary Information

**Additional file 1: Supplement 1.** Questions utilised in the cross-sectional study. **Supplement 2.** STROBE Statement—Checklist of items that should be included in reports of cross-sectional studies.

## Data Availability

All data generated or analysed during this study are included in this published article [and its supplementary information files].
