# Supplementary material for: Workplace factors associated with mental health of healthcare workers during the COVID-19 pandemic: an international cross-sectional study
Source: BMC Health Serv Res. 2021 Mar 21;21:262. doi: 10.1186/s12913-021-06279-6 (PMC7981382; doi:10.1186/s12913-021-06279-6)
Supplement: Supplementary file 1 — Additional file 1: Supplement 1. Questions utilised in the cross-sectional study. Supplement 2. STROBE Statement—Checklist of items that should be included in reports of cross-sectional studies. [file 12913_2021_6279_MOESM1_ESM.docx]

**SUPPLEMENT**

**Supplement 1. Questions utilised in the cross-sectional study**

**SECTION 1 – Demographic data**

- **Gender**
  - Female
  - Male
  - Prefer not to say
  - Other
- **Age**
  - 18-25
  - 26-33
  - 34-40
  - 41-48
  - 49-56
  - Over 56
- **Role**
  - Intern/Foundation Trainee
  - Senior House Officer (SHO)/Junior Resident
  - Registrar/Senior Resident
  - Consultant/Attending
  - General Practioner (GP)
  - Hospital management/administrative staff
  - Junior Nurse
  - Senior Nurse
  - Matron/Chief nurse
  - Medical Student
  - Nursing Student
  - Allied healthcare professionals (e.g. Occupational therapists, physiotherapists, dieticians)
  - Volunteer
  - Other
- **Which country are you based in?** (dropdown)
- **Which city are you based in?** (dropdown)
- **Have you been personally involved in the care of COVID-19 positive patients who have died?**
  - Yes
  - No
- **Have you been redeployed to help in a different specialty or area?**
  - Yes
  - No
- **If yes to the above question, which area/specialty have you been redeployed to?**
  - Intensive Care Unit (ICU)/Critical Care
  - Emergency Medicine
  - General Medicine
  - I have not been redeployed
  - Other
- **If redeployed, how satisfied did you feel about the amount and quality of training received for the new role? (If not redelpoyed, tick N/A)**
  - Not at all satisfied
  - Partly satisfied
  - Satisfied
  - More than satisfied
  - Very satisfied
  - I did NOT receive training before starting my new role
  - N/A

**SECTION 2**

- **During this outbreak, have you felt down, depressed, or hopeless?**
  - None of the time
  - Rarely
  - Some of the time
  - Often
  - All the time
- **To what extent do you agree or disagree with this statement: "I feel well supported in the area/specialty I am currently working in"**
  - Strongly disagree
  - Disagree
  - Neither disagree or agree
  - Agree
  - Strongly agree
- **Have you felt at risk psychologically by not being issued appropriate PPE?**
  - None of the time
  - Rarely
  - Some of the time
  - Often
  - All the time
- **To what extent do you feel that you have received mental wellbeing support during this COVID-19 outbreak?**
  - Never
  - Rarely
  - Sometimes
  - Very often
  - Always

Supplement 2. STROBE Statement—Checklist of items that should be included in reports of ***cross-sectional studies***

|  | Item No | Recommendation | Page No |
| --- | --- | --- | --- |
| **Title and abstract** | 1 | (*a*) Indicate the study’s design with a commonly used term in the title or the abstract | 1 |
|  |  | (*b*) Provide in the abstract an informative and balanced summary of what was done and what was found | 2 |
| Introduction | | | |
| Background/rationale | 2 | Explain the scientific background and rationale for the investigation being reported | 4-6 |
| Objectives | 3 | State specific objectives, including any prespecified hypotheses | 5-6 |
| Methods | | | |
| Study design | 4 | Present key elements of study design early in the paper | 7-10 |
| Setting | 5 | Describe the setting, locations, and relevant dates, including periods of recruitment, exposure, follow-up, and data collection | 7-8 |
| Participants | 6 | (*a*) Give the eligibility criteria, and the sources and methods of selection of participants | 7-8 |
| Variables | 7 | Clearly define all outcomes, exposures, predictors, potential confounders, and effect modifiers. Give diagnostic criteria, if applicable | 8 |
| Data sources/ measurement | 8* | For each variable of interest, give sources of data and details of methods of assessment (measurement). Describe comparability of assessment methods if there is more than one group | 8 |
| Bias | 9 | Describe any efforts to address potential sources of bias | 9 |
| Study size | 10 | Explain how the study size was arrived at | 7 |
| Quantitative variables | 11 | Explain how quantitative variables were handled in the analyses. If applicable, describe which groupings were chosen and why | 9 |
| Statistical methods | 12 | (*a*) Describe all statistical methods, including those used to control for confounding | 9 |
|  |  | (*b*) Describe any methods used to examine subgroups and interactions | 9 |
|  |  | (*c*) Explain how missing data were addressed | NA |
|  |  | (*d*) If applicable, describe analytical methods taking account of sampling strategy | 7 |
|  |  | (*e*) Describe any sensitivity analyses | 13-19 |
| Results | | | |
| Participants | 13* | (a) Report numbers of individuals at each stage of study—eg numbers potentially eligible, examined for eligibility, confirmed eligible, included in the study, completing follow-up, and analysed | 11    1 |
|  |  | (b) Give reasons for non-participation at each stage | N/A |
|  |  | (c) Consider use of a flow diagram | N/A |
| Descriptive data | 14* | (a) Give characteristics of study participants (eg demographic, clinical, social) and information on exposures and potential confounders | 11-13 |
|  |  | (b) Indicate number of participants with missing data for each variable of interest | NA |
| Outcome data | 15* | Report numbers of outcome events or summary measures | 11-19 |
| Main results | 16 | (*a*) Give unadjusted estimates and, if applicable, confounder-adjusted estimates and their precision (eg, 95% confidence interval). Make clear which confounders were adjusted for and why they were included | 11-19 |
|  |  | (*b*) Report category boundaries when continuous variables were categorized | N/A |
|  |  | (*c*) If relevant, consider translating estimates of relative risk into absolute risk for a meaningful time period | N/A |
| Other analyses | 17 | Report other analyses done—eg analyses of subgroups and interactions, and sensitivity analyses | 13-19 |
| Discussion | | | |
| Key results | 18 | Summarise key results with reference to study objectives | 19 |
| Limitations | 19 | Discuss limitations of the study, taking into account sources of potential bias or imprecision. Discuss both direction and magnitude of any potential bias | 22 |
| Interpretation | 20 | Give a cautious overall interpretation of results considering objectives, limitations, multiplicity of analyses, results from similar studies, and other relevant evidence | 23 |
| Generalisability | 21 | Discuss the generalisability (external validity) of the study results | 20,  21, 23 |
| Other information | | | |
| Funding | 22 | Give the source of funding and the role of the funders for the present study and, if applicable, for the original study on which the present article is based | 1 |
